# Contrasting Immunomodulatory Effects of Probiotic and Pathogenic Bacteria on Eastern Oyster, Crassostrea Virginica, Larvae

**DOI:** 10.3390/vaccines8040588

**Published:** 2020-10-06

**Authors:** Tejashree H. Modak, Marta Gomez-Chiarri

**Affiliations:** 1 Department of Cell and Molecular Biology, University of Rhode Island, Kingston, RI 02881, USA; tejashree@uri.edu; 2 Department of Fisheries, Animal and Veterinary Sciences, University of Rhode Island, Kingston, RI 02881, USA

**Keywords:** *Crassostrea virginica*, *Vibrio coralliilyticus*, larvae, oyster hatchery, probiotics, *Bacillus pumilus*, *Phaeobacter inhibens*, transcriptome

## Abstract

Several *Vibrio* spp. cause acute and severe mortality events in hatcheries where larvae of bivalve mollusks are reared, potentially leading to subsequent shortage of bivalve seed for the grow-out industry. In particular, strains of *Vibrio coralliilyticus* have been identified as a major cause of disease in Pacific, *Crassostrea gigas,* and eastern, *C. virginica,* oyster hatcheries in the USA of America. Probiotic bacteria are an inexpensive, practical, and natural method of disease control. Previous research shows that pretreatment of larval oysters with probiotic bacteria *Bacillus pumilus* RI06–95 (RI) and *Phaeobacter inhibens* S4 (S4) significantly decreases mortality caused by experimental challenge with the bacterial pathogen *V. coralliilyticus* RE22 (RE22). This study aims to characterize the immune response of 6–10-day-old eastern oyster larvae to experimental challenge with pathogen *V. coralliilyticus* RE22 and probionts RI and S4. Treatments included (a) pathogen and probiont exposure at a concentration of 5 × 10^4^ CFU per mL (~2500 bacterial cells per larva) for a duration of 6 h, (b) probiont exposure at the same concentration for a duration of 24 h, and (c) probiont RI daily treatment of larvae in the hatchery for 4, 11, and 15 days. Differential gene expression analysis compared pathogen or probiotic-treated transcriptomes to unexposed controls. Probiotic and pathogen treatment led to upregulation of transcripts coding for several immune pattern recognition receptors (PRRs) involved in environmental sensing and detection of microbes in oyster larvae. Larval oyster responses to pathogen RE22 suggested suppression of expression of genes in immune signaling pathways (*myd88, tak1, nkap*), failure in upregulation of immune effector genes, high metabolic demand, and oxidative stress that potentially contributed to mortality. On the other hand, the transcriptomic response to probiotic bacteria RI and S4 suggested activation of immune signaling pathways and expression of immune effectors (e.g., *Cv-spi2*, *mucins* and *perforin*-*2*). These key features of the host immune response to probiotic bacteria were shared despite the length of probiotic exposure, probiotic species, and the type of environment in which exposures were conducted. This study suggests that pre-exposure of eastern oyster larvae to probiotics for 6–24 h prior to pathogenic challenge leads to a robust and effective immune response that may contribute to protecting larvae from subsequent challenge with *V. coralliilyticus* RE22. This research provides new insights into host-microbe interactions in larval oysters that could be applied in the management of vibriosis in bivalve hatcheries.

## 1. Introduction

The *Vibrionaceae* constitutes a diverse bacterial family inhabiting a variety of ecological niches in aquatic environments. Several *Vibrio* spp. are well-known for their ability to cause disease in a broad range of marine, estuarine, and freshwater hosts, as well as in the terrestrial organisms that ingest vibrio-contaminated seafood or water [1,2,3]. These diseases, grouped under the generic name of vibriosis, have a significant economic and ecological impact, constraining the productivity of aquaculture and fisheries, affecting keystone species such as reef-building corals and bivalves, and placing a burden on public health, particularly in coastal regions [4,5,6].

Vibriosis is, in general, considered an opportunistic disease, affecting hosts at early life stages (e.g., larvae and juveniles) or when they are compromised (e.g., due to environmental stress or other diseases), therefore constituting an emerging problem due to stress caused by climate change [2,4,6]. In the case of bivalve mollusks (oysters, clams, scallops, mussels), vibriosis causes significant losses in hatcheries and nurseries that produce the larvae and seed supporting bivalve aquaculture, one of the most important sectors of aquaculture [7,8,9,10,11]. Several *Vibrio* spp. have been isolated from mortality outbreaks in bivalve hatcheries and nurseries, including *V. aestuarianus, V. coralliilyticus, V. splendidus, V. tapetis, V. tasmaniensis,* and *V. tubiashii,* to name a few. Strains of *V. coralliilyticus,* in particular, have been isolated from multiple outbreaks of disease in both Pacific oyster, *Crassostreae gigas,* and eastern oyster, *C. virginica,* hatcheries [7,12,13] and have been demonstrated to cause larval mortality in experimental infections [14,15,16]. Despite the diversity of *Vibrio* spp. able to cause disease in bivalve hatcheries, the clinical signs of vibriosis in larval bivalves are generally similar. Vibrio larval infection is dramatically rapid in progression and characterized with signs of reduced motility and feeding, bacillary necrosis, swarming of bacteria in the tissues and around the moribund larvae, and death within hours of infection [17]. Recent studies suggest that bivalve larvae and juveniles are unable to mount an effective immune response against vibrio challenge, as seen in mussel larvae in response to *V. coralliilyticus* [18] and in Pacific oysters in response to *V. crassostreae* and *V. tasmaniensis* [19,20].

Practices to reduce mortality due to bacterial disease in aquaculture systems include treatment with antibiotics and disinfection of seawater. Water treatment, however, is expensive and could be toxic to the larvae if not done properly, while antibiotic treatment raises environmental and human health concerns [21,22,23]. Therefore, alternative methods need to be developed to manage good larval rearing environment and to control bacterial diseases in bivalve shellfish hatcheries [24]. Probiotics are defined as live microbial food supplements that confer a health benefit on the host [25]. Probiotics are known to benefit the host by a variety of means, including production of antimicrobials, improving water quality, enhancing the immune responses of host, and competing for space with pathogenic bacteria [4,26,27,28,29]. The benefits of probiotics have already been shown in Pacific [30,31,32] and eastern [33,34] oysters. Pretreatment of larval and juvenile *C. virginica* with probiotic bacteria *Phaeobacter inhibens* S4 (referred to as S4) and *Bacillus pumilus* RI06–95 (RI) for at least one day improves eastern oyster survival after experimental challenge with the bacterial pathogens *Aliiroseovarius crassostreae* and *Vibrio coralliilyticus* RE22 (RE22) [33,34]. The mechanisms of action of these probiotics are likely complex. Similarly, to other *Phaeobacter* spp., probiont S4, a Gram-negative organism, is a strong biofilm former that produces the antibiotic tropodithietic acid (TDA) [35,36,37,38]. Although these two mechanisms are involved in protecting oysters and other organisms from bacterial infection, mutants of S4 unable to produce TDA and with decreased ability to produce biofilms still provide some level of protection [38], suggesting that other mechanisms are also potentially involved, including interference of quorum sensing signaling in the pathogen [39]. Probiont RI is a Gram-positive organism that produces the antibiotic amicoumacin, but this antibiotic does not inhibit the growth of RE22 in an in vitro assay, indicating that other mechanisms of action are also likely involved in RI’s protection of larvae against bacterial challenge [33]. There is growing evidence that probiotics show immunomodulatory effects in fish and shellfish [40,41]. Our hypothesis is that pre-treatment of oyster larvae with probionts RI and S4 may cause an activated immune state in eastern oyster larvae that contribute to protection against the pathogenic effects of RE22.

Based on the large economic impact of vibriosis on bivalve hatcheries worldwide [7,8,9,10,11], more research is needed to understand the variety of mechanisms used by vibrio species to evade immunity and cause disease in bivalve host species. More research is also needed on the development of effective tools, such as probiotics, to manage vibriosis in bivalve hatcheries. The goal of this study is to characterize the immune response of *C. virginica* larvae to experimental challenge with the bacterial pathogen *V. coralliilyticus* RE22 and two probiotic bacterial species, *Phaeobacter inhibens* S4 and *Bacillus pumilus* RI06–95, using transcriptomic analysis. This research provides hypotheses on possible strategies used by RE22 to overcome larval immune defenses and increases our understanding on the role of immunomodulation as a potential mechanism of action of probiotics in providing protection against vibriosis. This knowledge will aid in the future development of solutions to control disease and design better management practices for bivalve hatcheries.

## 2. Materials and Methods

### 2.1. Bacterial Cultures

The pathogen, *Vibrio coralliilyticus* RE22 [15] and probiotics, *Bacillus pumilus* RI06–95 and *Phaeobacter inhibens* S4 [33] were maintained and stored in 50% glycerol stocks at −80 °C until use. Inocula from freezer stocks were plated on yeast peptone with 3%NaCl (YP30; 5 g·L^−1^ of peptone, 1 g·L^−1^ of yeast extract, 30 g·L^−1^ of ocean salt, Instant Ocean) agar plates for 1 d (for RE22) and 2 d (for RI06–95 and S4), then transferred to 5 mL of YP30 broth incubated at 25 °C on a shaker (134 rpm) for 1 d (for RE22) and 2 d (for RI and S4). Cultures were washed using Artificial Filtered Sterile Seawater (AFSW, 28–30 psu salinity) twice by centrifugation at 12,000 rpm (13,100 rcf) for 10 min. The optical density at 550 nm (OD_550_) was measured and the stock was diluted such as to obtain target concentrations based on a previously determined relationship between colony forming units (CFU) and OD_550_ [33].

### 2.2. Oyster Larvae

Eastern oyster, *C. virginica,* larvae were obtained from shellfish hatcheries on the east coast of USA of America. Larvae are most susceptible to *V. coralliilyticus* RE22 infection during the first two weeks of life [14,16]. Larvae 6–10 days old (veliger stage), selected from those passing through a 175 µm sieve but retained on a 100 µm sieve, were collected at the hatchery and shipped overnight to the laboratory on a wet filter in a box filled with ice blocks. Upon arrival, larvae were washed with AFSW (28–30 psu salinity, room temperature—RT, 22–23 °C) on top of a 40 µm nylon mesh and placed in stock containers containing AFSW. Larvae were acclimatized to the laboratory environment for 24 h prior to the experiments. Larval density (larvae mL^−1^) of the stock was determined using a Nikon E200 microscope (Nikon).

### 2.3. Effect of V. coralliilyticus RE22 on Survival of C. virginica Larvae

**Figure 1:**
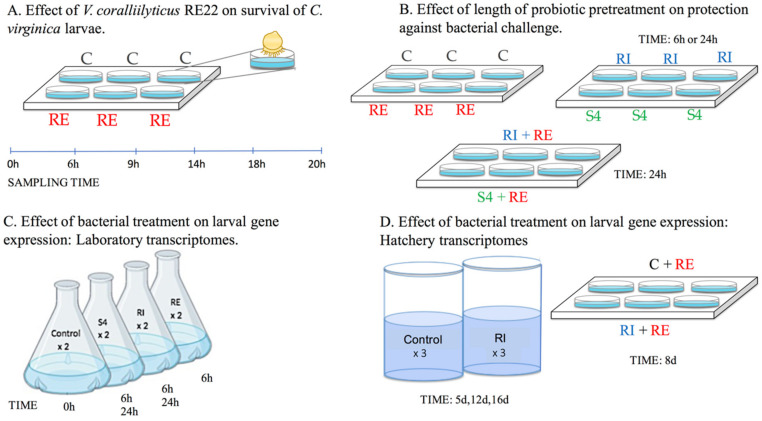
Schematic showing experimental design for the larval survival and transcriptomic experiments performed. Labels (**A–D**) represent individual experimental designs. Treatments include probionts *Phaeobacter inhibens* S4 and *Bacillus pumilus* RI0-695, denoted as S4 and RI, respectively, pathogen *V. coralliilyticus* RE22, denoted as RE, and control denoted as C.

In order to understand the rate of progression of bacterial disease (vibriosis) and determine the optimal sampling time point for the transcriptome study, *C. virginica* larvae were experimentally challenged with *V. coralliilyticus* RE22 (Figure 1A).

Larvae (~100–175 µm in size) were distributed in wells of a 6-well plate (~100 per well) with 5 mL AFSW and maintained at RT with gentle rocking. Treatments (control and RE22 challenge) were each conducted in triplicate. Larvae were challenged by addition of a target final concentration of 5 × 10^5^ CFU of RE22 per mL of water (~2.5 × 10^4^ vibrio cells per larva) in each well mixed with 1 mL of instant algae Shellfish Diet 1800^TM^ (20,000 cells mL^−1^; Reed Mariculture Inc., San Jose, CA. USA) in order to promote pathogen ingestion. Larval mortality was recorded at 6, 9, 14, 18, and 20 h post addition of RE22 by evaluation of active swimming and/or gut and cilia movement using a Nikon E200 microscope (Nikon) [33].

### 2.4. Effect of Length of Probiotic Pretreatment on Protection against Pathogen Challenge

In order to determine the effect of pre-treatment of larvae with probiotics on protection against challenge with the bacterial pathogen *V. coralliilyticus* RE22, ~100 larvae (~100–175 µm) were placed in each well of a 6-well plate in 5 mL of AFSW and incubated with 5 × 10^4^ CFU mL^−1^ of probionts S4 or RI for either 6 h or 24 h (Figure 1B) prior to bacterial challenge with 5 × 10^5^ CFU mL^−1^ of RE22. Treatments were each conducted in triplicate. Larval survival was determined 24 h after RE22 challenge (time at which the survival of control challenged larvae ranged between 25 and 50%, while non-challenged larvae ranged between 80–100%) using previously described methods [33]. Survival rate was calculated as follows: Survival rate (%) = 100 × (number of live larvae/total number of larvae). One-way analysis of variance (ANOVA) on transformed data was used to determine significance between treatments and Tukey’s multiple comparison tests were used for post-hoc pairwise comparisons (*p* < 0.05) [34]. The relative percent survival (RPS) of probiotic pretreatment (treatment) compared to the challenged larvae (control) was calculated using the formula:RPS (%) = (1 − (% Mortality treatment / % Mortality control)) × 100 [33].

### 2.5. Effect of Bacterial Treatment on Larval Gene Expression: Laboratory Transcriptomes

For biological replicates, three independent experiments were performed using larvae of the same size range (~100–175 µm) from three different hatcheries (designated as K, M, and V), to account for variability in larval genetics and hatchery environments (Figure 1C). Two parallel exposures were performed with each set of larvae:

a) Transcriptome analysis:

Larvae were distributed into sterile tissue culture flasks (~10,000 per flask) in 500 mL AFSW and kept on a shaker with gentle shaking at ~50 rpm at room temperature. Larvae were acclimatized to the experimental set up for an additional 24 h prior to treatment. Each treatment (control, probiont RI and S4, and pathogen RE22) was conducted in duplicate to serve as technical replicates. Bacterial treatments were performed by adding them at a concentration of 5 × 10^4^ CFU mL^−1^ culture water (~2500 bacterial cells per larva).

**Figure 2:**
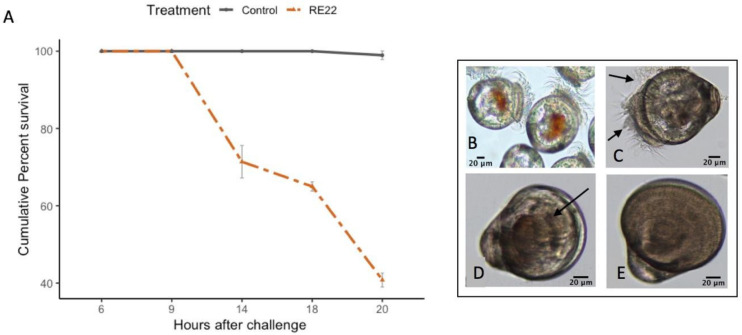
Effect of challenge with *Vibrio coralliilyticus* RE22 on *C. virginica* larvae. (**A**) Cumulative percent mortality +/− standard error in oyster larvae after 6–20 h of challenge with 5 × 10^5^ CFU mL^−1^ of RE22 (*n* = 6 experiments). Mortality was first observed at 14 h after challenge, and rapidly increased thereafter. (**B**) Actively swimming healthy control larvae. (**C**) Larvae showing clumping of the cilia 6 h after challenge. (**D**) Moribund larva with retracted cilia showing reduced movement at 9 h after challenge. (**E**) Dead larva with empty shells at 14 h after challenge.

Control and bacterial-treated larvae were collected 6 h (for RE22, RI, and S4) and 24 h (for probiotics only) post-exposure. These time points were selected in order to determine: a) the early immune response of the larvae to the pathogen before onset of mortality at 9 h (Figure 2) the effect of probiotic treatment on larval responses immediately prior to RE22 challenge.

Larvae were aspirated gently from the incubation flasks using a 100 mL serological pipette, carefully avoiding to collect the bottom 25 mL (non-swimming and dead larvae). Larvae were filtered through a 40 µm sterile filter, washed with 2 mL of AFSW, followed by a wash using 2 mL of RNAlater™ (Ambion, Inc., Foster City, CA, USA), aspirated from the 40 µm filter using a pipette, and placed in RNase-free microfuge tubes. Larvae were held at 4 °C for 24 h in RNAlater™ followed by storage at −20 °C until RNA extraction.

b) Survival analysis:

To confirm the effect of treatment on larval survival after RE22 challenge, oyster larvae (~100, 100–175 µm) were placed in each well of a 6-well plate in 5 mL of AFSW and triplicate wells per treatment were incubated at room temperature with 5 × 10^4^ CFU·mL^−1^ of probionts S4 or RI for 6 or 24 h prior to bacterial challenge with 5 × 10^5^ CFU mL^−1^ of RE22. Larval survival was determined 6 h (for RE22 and control) and 24 h (for all groups) after challenge as described above.

### 2.6. Effect of Bacterial Treatment on Larval Gene Expression: Hatchery Transcriptomes

Adult eastern oysters were spawned and larvae were reared in 100 L conical tanks filled with filtered and UV treated seawater (20–24 °C temperature; 28–30 psu salinity) at the Blount Shellfish Hatchery, Roger Williams University, RI following previously described procedures [34]. Briefly, larval oysters were distributed into 100 L conical tanks 1 d after fertilization to a density of 8–10 larvae per mL of tank water under static conditions with gentle aeration and fed live microalgae daily from a microalgae production greenhouse. Treatments (three tanks each) included control and probiont RI (10^4^ CFU·mL^−1^). Probiotic stocks were mixed with algal food and added daily for 15 days at the time of feeding. Full tank water changes were performed every 2 days to maintain water quality and determine larval growth and survival (Figure 1D).

a) Transcriptome analysis:

Larvae for hatchery transcriptomes were collected at three time points: 5, 12, and 16 days post-fertilization (4, 11, and 15 days of treatment) from probiotic-treated (HT_RI) and control tanks (HT_C). Tanks were drained onto a sieve with suitable pore size (75–150 µm depending on the age of the larvae) at the time of collection and washed gently with clean seawater. Using a serological pipette, larvae were aspirated gently from the sieve and collected in RNase free microfuge tubes with RNAlater™ and stored at −80 °C until RNA extraction.

b) Survival analysis:

A subsample of larvae was collected from each tank on day 8 post-fertilization to determine the effect of exposure to probiont RI in the hatchery on protection against a laboratory challenge with pathogen RE22. Levels of protection were determined using the methods described above, with the following modifications: a subset of larvae from each tank (3 × HT_C and 3 × HT_RI) were placed in triplicate wells of 6-well plates containing 5 mL of AFSW per well, acclimated for a few hours, and then directly challenged with *V. coralliilyticus* RE22 at a dose of 5 × 10^5^ CFU·mL^−1^.

### 2.7. RNA Extraction, cDNA Prep, and Sequencing

Tri-reagent™ (Sigma-Aldrich, St. Louis, MO, USA) was used for extracting total RNA from all the samples following manufacturer’s instructions. RNA extracts were DNase treated using a DNA-free™ DNA removal kit (Ambion Inc., Foster City, CA, USA) and purity and concentration of RNA was assessed using a Nanodrop 8000 spectrophotometer (Thermo Scientific, Wilmington, DE, USA). RNA from the two technical replicates in each experiment was pooled at equimolar concentration. The quality and quantity of the pools were assessed using Agilent 2100 Bioanalyzer (Agilent, Santa Clara, CA, USA) and High Sensitivity D1000 ScreenTape® (Agilent, Santa Clara, CA, USA). For the laboratory experiments, RNA samples were selectively enriched for poly-A containing mRNA and cDNA libraries were prepared using the PrepX RNAseq library Prep Kit (Takara Bio USA, Inc, Mountain View, CA, USA). Samples were sequenced on Illumina HiSeq platform with 2 × 125 reads at a targeted sequencing coverage of 20–30M per sample at the Harvard University, FAS Center for Systems Biology, Boston, MA. For the hatchery experiments, cDNA libraries were generated using random hexamer priming of total RNA and sequenced on Illumina HiSeq platform with 2 × 150 reads and sequencing coverage of 50–70M per sample at the McDonnell Genomics Institute, Washington University School of Medicine, MO.

### 2.8. Assembly, Annotation, and Differential Expression Analysis

Raw reads obtained from sequencing were filtered, trimmed, and adapters were removed using bbduk program in BBTools suite from Joint Genome Institute and viewed in FASTQC [42]. Processed reads were aligned to *C. virginica* reference genome (version 3.0, GenBank GCA_002022765.4) via HISAT2 2.1.0 [43] and assembly was performed using Stringtie [44] using default parameters. To compare the depth of sequencing across all samples, preseq package was used [45]. Differential gene expression analysis was performed by comparing transcript counts between probiotic (RI and S4; replicates K, M, V) or pathogen treatments (RE22; replicates K, M, V) vs the control (replicates K, M, V) using DESeq2 [46]. This design controls for batch effects due to larval environment at the hatchery, genetic background of the larvae, and differences in age (all larvae collected were in the veliger developmental stage). For hatchery transcriptomes, each of the days (5, 12, and 16 post-fertilization) were considered as biological replicates and an overall comparison of treatment vs control was conducted. Transcript counts for each replicate were used to determine levels of expression for DEGs in each individual time point. This analysis design only allowed for the most conservative estimates and only showed differentially expressed genes in all the biological replicates (time points) responding to treatment (i.e., RI treatment effect). Transcripts with Benjamini-Hochberg adjusted *p*-value ≤ 0.05 and log fold change of ≥ 2 or ≤ −2 were considered significantly differentially expressed. Annotation for differentially expressed genes (DEGs) was performed by mapping to NCBI protein non-redundant (NR) database using BLASTx [47] with an e-value cutoff of 1e^−3^ and hit number threshold of 20. Mapping DEGs to GO terms was conducted using BLAST2GO v4.1.9 [48] and functional enrichment was done using topGO [49] with default parameters. Significantly enriched GO terms were obtained by using Fishers exact test (*p* ≤ 0.01).

The raw sequences generated for this study can be found in the NCBI Short Read Archive under BioProject no. PRJNA603627 for all laboratory transcriptomes and under BioProject no. PRJNA376014 for all hatchery transcriptomes.

## 3. Results

### 3.1. Rapid Mortality in C. virginica Larvae after Challenge with Pathogen V. coralliilyticus RE22

Oyster larvae exposed to 5 × 10^5^ CFU mL^−1^ of *V. coralliilyticus* RE22 showed a rapid decline in survival between 9 and 14 h after challenge, reaching about 40% survival at 20 h after challenge (Figure 2A). Larvae showed a normal appearance before challenge (Figure 2B). At 6 h after challenge, many larvae showed clumping of the cilia by bacteria, leading to reduced motility and feeding (Figure 2C). Moribund larvae collected 9 h after challenge showed larvae with cilia drawn in, reduced activity, and the presence of bacteria and debris aggregates inside the shells (Figure 2D) and dead larvae at 14 h showed no visible sign of live organs or tissue inside their shell, just bacterial aggregates and debris (Figure 2E).

### 3.2. Pretreatment of Larvae with Probionts RI and S4 for 6 or 24 h before Challenge Conferred Protection against Pathogen RE22

As seen in previous research [33], pretreatment of larvae with probionts *B. pumilus* RI and *P. inhibens* S4 for 24 h significantly reduced mortality caused by a subsequent challenge with the bacterial pathogen *V. coralliilyticus* RE22 from 39–43% to 72–77% (with RI-pretreated larvae, *p* < 0.05, ANOVA) and 68–70% (with S4-pretreated larvae; *p* < 0.05, ANOVA), signifying a relative increase in percent survival (RPS) in probiotic treated larvae compared to non-treated larvae of around 40% (Table 1).

A shorter duration of S4 or RI pretreatment (6 h) led to an increase in larval survival after bacterial challenge from 29–78% with RI-pretreated larvae to 36–72% with S4-pretreated larvae, but the levels of protection conferred were highly variable and not significant in one of the three experiments performed (see Table 1 for RPS), suggesting variability between larval batches on the kinetics of the response to the probiotics. Larvae treated daily with probiotics for 7 days in the hatchery, and then exposed to RE22 for 24 h in the laboratory, showed an increase in survival from an average of 42% (control tanks) to 63% (RI-treated tanks; see Table 1 for RPS).

### 3.3. Transcriptome and Differential Gene Expression Analysis Statistics

Depth of sequencing for all laboratory transcriptomes ranged from 14–39M paired end reads, whereas HT_RI transcriptomes ranged from 50–73M reads (Supplementary Table S1). The alignment rate to the C. virginica reference genome using HISAT2 ranged from 83–89% for laboratory transcriptomes and from 53–94% for HT_RI and HT_C transcriptomes (Supplementary Table S1). The lower percentage of mapped reads to the eastern oyster genome seen in some of the hatchery transcriptomes was due to the presence of microbial reads. Sequencing saturation curves for all hatchery transcriptomes were comparable between treatments and close to full saturation, indicating enough depth of sequencing such that all but the rarest (least abundant) transcripts would be represented in the transcriptomes. The percentage of oyster transcripts annotated was 98%.

**Figure 3:**
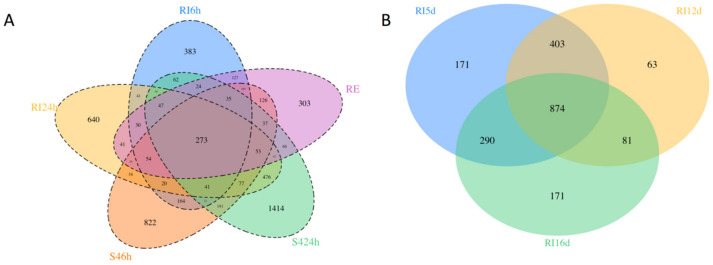
Comparison of differentially expressed genes in probiotic treatments. (**A**) Venn Diagram of shared and unique differentially expressed genes for larvae exposed to *Vibrio coralliilyticus* RE22 (6 h), probiotic (*Bacillus pumilus* RI0-695 and *Phaeobacter inhibens* S4) at 6 h and 24 h in laboratory experiments (*n* = 3). (**B**) Venn diagram comparing number of differentially expressed genes in probiont RI pre-treated larvae at 5, 12, and 16 d post-fertilization (4, 11, and 15 d of RI exposure) as compared to the respective untreated control in a hatchery (*n* = 3 tanks per treatment).

The number of differentially expressed genes (DEGs) in each of the pair-wise (control vs. treatment) comparisons ranged from 1461 (RE22 at 6 h) to 2892 (S4 at 24 h; Supplementary Table S2). Supplementary dataSupplementary data Tables S3–S8 include the annotation and log fold change values for DEGs for all comparisons. Comparison of the number of shared and unique DEGs across all laboratory treatments including pathogen (RE22 6h) and probiotic at both time points (RI and S4; 6 and 24 h; Figure 3A) showed more unique transcripts in larvae exposed to probiotic treatments at any time point (from 7% of all DEGs for RI6h to 24% for S424h) than in larvae exposed to the pathogen for 6 h (5%). Only 5% of the DEGs were shared between all bacterial treatments and time points, with 29% shared by the two probiotics (for both time points combined). Larvae treated with probiotics for 24 h in the laboratory yielded more DEGs than at 6 h (Figure 3A and Supplementary Table S2), suggesting a build-up of the response between 6 and 24 h. A comparison between hatchery transcriptomes (HT_RI) at all time points (5, 12, and 16 d post-fertilization; Figure 3B) showed a higher number of DEGs at 5 than 12 and 16 d, with 43% of the DEGs being shared between time points.

### 3.4. Gene Ontology Enrichment Analysis Showed an Enrichment in Genes Involved in Metabolism in Larvae Treated with RE22, but Not in Probiotic Treated Larvae

A Gene Ontology (GO) term enrichment analysis was performed on all the annotated differentially expressed transcripts in response to each treatment (Supplementary Table S9). In RE22 exposed larvae, there were 18 biological processes significantly enriched (*p* < 0.05) that mainly belonged to metabolism and signaling, but none related to immunity; 17 metabolic functions were significantly enriched (*p* < 0.05) including “receptor activity,” “molecular transducer activity,’ and “kinase activity.” Transcripts annotated to the “membrane” category in the GO Cellular component class were also significantly enriched. In contrast, S4 treatment showed significant enrichment only in processes related to activation of receptors and signaling pathways. No GO terms were significantly enriched among DEGs from the comparison between the control and probiotic RI treatment in the laboratory, but the hatchery exposure to RI led to enrichment in several categories, most of them related to location of molecules/organelles/cells (Supplementary Table S9). The HT_RI transcriptomes also showed significant levels of enrichment in more categories, sharing with S4_24 h an enrichment in transcripts annotated to the term “cytoskeletal organization.”

### 3.5. Contrasting Effects of Treatment with Probionts RI and S4 and Pathogen RE22 on Larval Immunity

**Figure 4:**
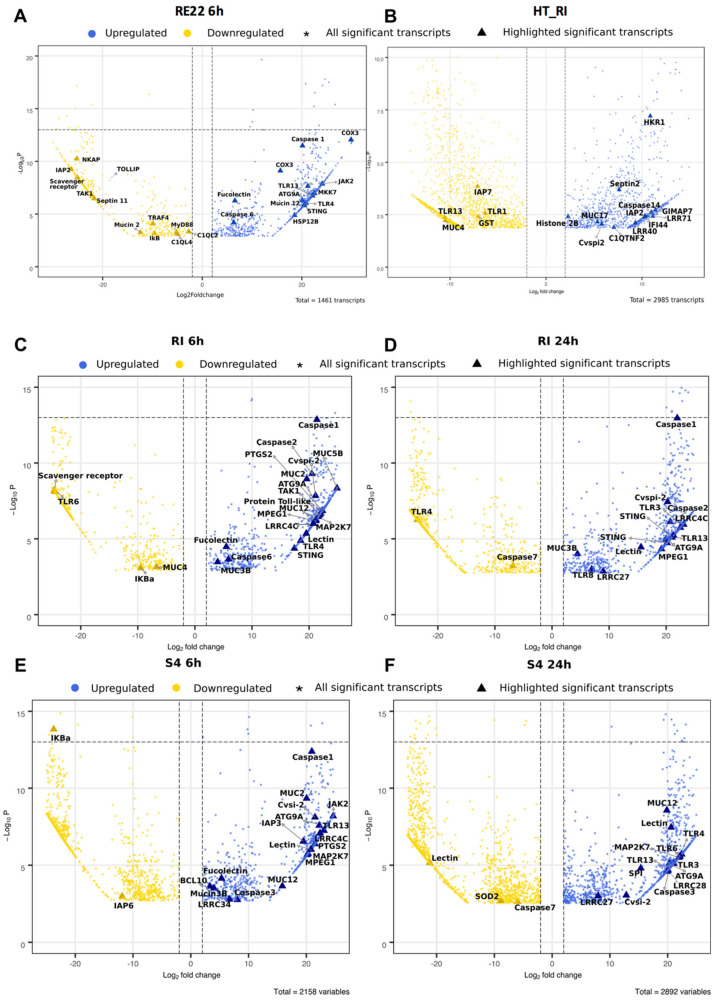
Effect of bacterial treatments on oyster larval gene expression. Volcano plot representation of significantly differentially expressed genes in each treatment versus control *C. virginica* larvae (*p* < 0.05). Dots mark the genes with highly decreased (gold) or increased (blue) expression in (**A**) *Vibrio coralliilyticus* RE22, (**B**) probiotic *Bacillus pumilus* RI06–95 in hatchery, (**C,D**) probiotic *Bacillus pumilus* RI06–95 in laboratory, and (**E,F**) probiotic *Phaoebacter inhibens* S4 exposed larvae compared to control. Immune-related differentially expressed genes are highlighted by a larger symbol (∆) in the same color scheme. The x-axis shows log2fold-changes in expression and the y-axis the negative log of *p* value of a gene being differentially expressed. Dashed lines indicate thresholds (+/− 2 change in log_2_ fold change in gene expression for vertical, 12 −log_10_P significance for horizontal).

**Figure 5:**
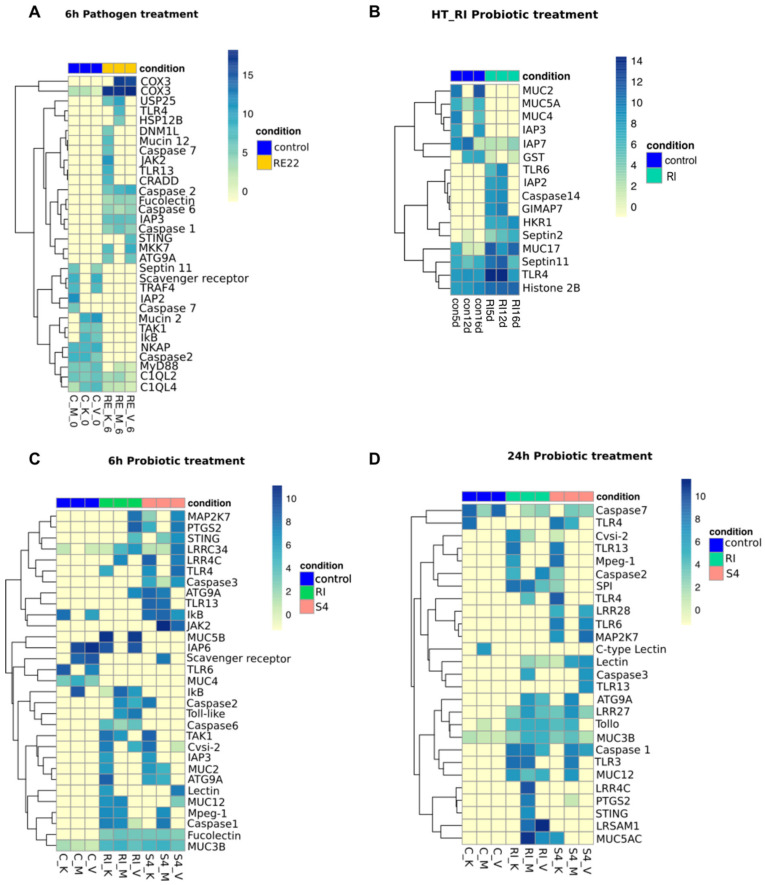
Differential expression of immune-related genes between control and pathogen or probiotic treated larvae. Heatmap and hierarchical clustering of selected genes based on normalized read counts. Each column represents one sample showing the intensity of expression profile per transcript. The colors denote the relative standing of the read count data with yellow indicating lower than the genes’ average across all samples while blue indicates higher than the average. The expression profiles are shown for control condition (C_M, C_K, C_V) for laboratory treatments and (Con5d, Con12d, Con16d) for hatchery treatments and (**A**) pathogen RE22 exposed larvae (RE_M, RE_K, RE_V) (**B**) probiont *Bacillus pumilus* RI06–95 for hatchery treatment (RI5d, RI12d, RI16d) (**C**) 6 h probiont *Bacillus pumilus* RI06–95 (RI_M, RI_K, RI_V) and *Phaoebacter inhibens* S4 exposed larvae (S4_M, S4_K, S4_V) for laboratory treatment (**D**) 24 h probiont *Bacillus pumilus* RI06–95 (RI_M, RI_K, RI_V) and *Phaoebacter inhibens* S4 exposed larvae (S4_M, S4_K, S4_V) as obtained from DESeq2 analysis.

A more detailed analysis of the effect of bacterial exposure on differential gene expression of immune-related genes in oyster larvae was performed, illustrated in volcano plots (Figure 4) and heat maps (Figure 5). Despite variability in the immune response between batches of larvae [34], some significant patterns in immune responses were observed. Overall, larvae treated with RE22 for 6 h showed a similar number of upregulated and downregulated immune-related transcripts (Figure 4A and Figure 5A), while probiotic treatment led to mostly upregulation of immune-related transcripts (Figure 4B–F and Figure 5B–D).

#### 3.5.1. Pattern Recognition Receptors (PRRs)

Exposure of larvae to either pathogen or probiotics for 6 h led to an upregulation of transcripts coding for fucolectin and one Toll-Like Receptor (TLR), TLR4 (the “shared” PRR response to bacterial exposure). Larvae exposed to pathogen RE22 and probiont S4 (both Gram-negative bacteria), but not probiont RI (a Gram-positive), also shared the upregulation of *tlr13*. Exposure to probionts RI and S4 for 6 h led to a broader response than exposure to RE22, with upregulation of three additional transcripts coding for leucine rich receptors LRRC34 (leucine-rich repeats) and LRR4C and a lectin. Transcripts for a few additional PRRs were shown to be upregulated in probiotic-treated larvae after 24 h, including 74ALRR27 (in both RI and S4) and LRR28 (in S4). Probiotic (RI and S4) exposure for 24 h also led to upregulation of *tlr3*, a cytosolic receptor that detects non-self nucleic acids. Exposure to pathogen led to significant downregulation of gene expression for transcript annotated to three PRRs (a scavenger receptor, and two complement C1q-like proteins), while two other PRR transcripts were downregulated in response to probiotic exposure (a scavenger receptor for S4 and *tlr6* for both S4 and RI at 6 h). Downregulation of *tlr6* in S4-treated larvae for 6 h was followed by upregulation at 24 h (Figure 4 and Figure 5).

#### 3.5.2. Immune Signaling Pathways

Consistent with the observation that probiotic treatment led to PRR transcript upregulation, several transcripts involved in TLR signaling pathways were differentially expressed upon probiotic treatment suggesting activation of the NF-kB and MAP kinase (MAPK) pathways. These include upregulation of transcripts for activator B-cell lymphoma/leukemia 10-like (*bcl10;* S46h) and downregulation of inhibitor NF-kappa-B inhibitor alpha-like isoform X1 (*ikBa;* RI)) for the NF-kB pathway and upregulation of dual specificity mitogen-activated protein kinase kinase 7-like (*map2k7;* RI, S4), and mitogen-activated protein kinase kinase kinase 7-like (*tak1;* RI, S4) for the MAPK pathway (Figure 4B–F and Figure 5B,C). 

Interestingly, in larvae treated with RE22, and despite upregulation of *tlr4* and *tlr13*, transcripts related to the TLR signaling pathway, including myeloid differentiation primary response protein MyD88-like (*myd88*), TNF receptor-associated factor 4-like (*traf4*), and *tak1* showed downregulation (Figure 4A and Figure 5A). In addition, the genes coding for several members of the NF-kB pathway were downregulated upon RE22 challenge, including those for NF-kappa-B-activating protein-like *(nkap)* and *ikBa*. A transcript for toll-interacting protein-like (*tollip*, an inhibitory adaptor protein that interacts with several TLR signaling pathway components) was downregulated. In contrast to the downregulation of key molecules of the canonical NF-kB pathway, the gene coding for an essential component of the MAPK signal transduction pathway, *mkk7* (dual specificity mitogen-activated protein kinase kinase 7-like), was upregulated.

#### 3.5.3. Immune Effectors

Probiotic treatment in the laboratory led to modulation of gene expression for three types of major immune effectors: serine protease inhibitors (*cvspi2, spi*), mucins, both secreted gel-forming mucins (*muc2, muc5a, muc5b, muc19)* and cell surface mucins (*muc3b, muc4, muc12*), and macrophage-expressed gene 1 protein-like (*mpeg1/perforin-2*) (Figure 4B–F and Figure 5B,C). Larvae exposed to RI in the hatchery also showed a complex pattern of mucin gene expression, with transcripts coding for several mucins showing upregulation (*muc17, hkr1*), and others downregulation (*muc2, muc4, muc5a*). Hatchery larvae also showed upregulation of a transcript for the antimicrobial histone H2B. Conversely, and consistent with the downregulation of transcripts involved in immune signaling pathways observed in RE22 treated larvae, the only immune effector differentially expressed in response to RE22 as compared to control were two types of transcripts related to mucin (*muc2, muc12).*

#### 3.5.4. Other Immune Molecules

Several transcripts related to antiviral pathways including stimulator of interferon genes protein-like (*sting;* all bacterial treatments) and some members of the JAK-STAT pathway (*jak2* (tyrosine-protein kinase JAK2-like)*;* S4 and RE22) were upregulated in response to bacterial exposures (Figure 4 and Figure 5). Differentially expressed transcripts corresponding to ubiquitin carboxyl-terminal hydrolase 25-like isoform X3 (*usp25*) were only seen in response to RE22. Moreover, several transcripts related to programmed cell death pathways were shown to be differentially expressed in larvae exposed to pathogen or probionts RI and S4 as compared to control larvae. These included upregulation of transcripts corresponding to autophagy related gene *atg9a* (autophagy-related protein 9A-like) and modulation (a combination of upregulation and downregulation, depending on the treatment and time) of several transcripts associated with the apoptosis pathway coding for death domain-containing protein CRADD-like (death domain-containing protein CRADD-like), initiator and executioner caspases, GTPase of the immune-associated protein 7 (GIMAP7, only in response to RI exposure in the hatchery), and several types of baculoviral inhibitor of apoptosis (IAP) repeat-containing proteins (Inhibitor of apoptosis protein) (Figure 4 and Figure 5). In particular, expression of *caspase1,* a key component of the inflammasome, was highly and consistently upregulated in all three bacterial treatments (Figure 4). Moreover, prostaglandin G/H synthase 2-like (PTGS2), important in inflammation, was also highly upregulated in all probiotic treatments except S4_24h.

#### 3.5.5. Gene Expression Patterns Show Signs of Metabolic Demand and Stress in RE22 Exposed Larvae

Multiple transcripts annotated as *cox3* (cyt oxidase c subunit III (mitochondrion))*, hspa12a* (heat shock proteins)*,* and *hspa12b,* which are involved in metabolism and stress, were seen as differentially expressed in all bacterial treatments (Supplementary Tables S3–S8). However, RE22 exposed larvae showed consistent upregulation of these genes, especially in *cox3* (Figure 4A and Figure 5A), whereas probiotic treatments showed a complex response, with both up- and downregulation of multiple transcripts depending on probiotic type or time of exposure (listed in Supplementary Tables S3–S8). In addition, transcripts coding for antioxidant enzymes, generally expressed to protect the host from a successful respiratory burst response to bacterial exposure, were not detected among significant DEGs in RE22 challenged larvae. In contrast, transcripts annotated as antioxidant enzymes were downregulated in probiotic treated larvae but not RE22 challenge, including transcripts coding for superoxide dismutase (SOD) and glutathione peroxidase 7-like (GPX7) that were downregulated in S4 (24h), and transcripts annotated as glutathione S-transferase (GST) downregulated in RI (6h and HT_RI) and S4 (24 h).

## 4. Discussion

**Figure 6:**
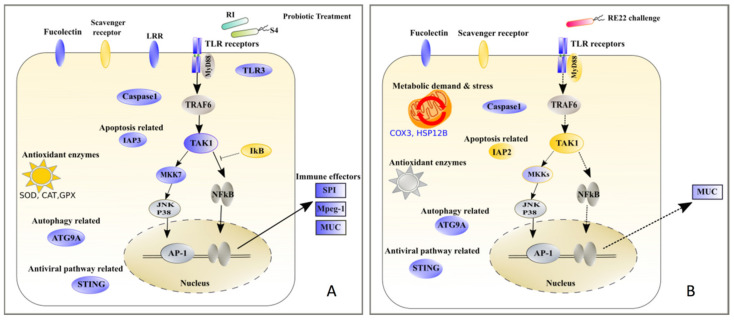
Summary of major impacts of challenge with *Vibrio coralliilyticus* RE22 and probiotic treatments with *Bacillus pumilus* RI06–95 and *Phaoebacter inhibens* S4 on *Crassostrea virginica* larval immune responses. (**A**) Exposure of larvae to probionts S4 and RI induced the expression of a large variety of immune genes, suggesting a strong immune response comprising pathogen recognition through a variety of PRRs with upregulation of several TLRs, lectins, and LRRs, activation of immune signaling pathways (e.g., TLR, NF-kB, MAPK, JAK-STAT and cGAS-STING), and upregulation of an arsenal of immune effectors (mucins, serine protease inhibitors, and perforin-2). DEGs also suggest modulation of cell death pathways and inflammation. (**B**) In contrast, in response to challenge with RE22, only a few immune receptors were upregulated, and several signaling molecules in the NF-kB pathway were downregulated (e.g., *myd88* and *tak1*), suggesting inactive immune signaling pathways resulting in lack of immune effectors. Upregulation of stress-related genes *cox3* and *hsp12a* suggest increased metabolic demand. Response showed absence of transcripts coding for antioxidant enzymes to combat oxidative stress. Purple represents gene upregulation, gold downregulation, and grey no change in expression.

The pathogen *V. coralliilyticus* RE22 and other pathogenic vibrios have been identified as a major threat to bivalve larvae worldwide [17], including *Crassostrea virginica,* a bivalve species of major commercial and ecological value in the Atlantic Coast of North America. A better understanding of the array of mechanisms used by different vibrio species to cause disease in a variety of shellfish species is needed in order to develop targeted tools for disease management, such as probiotics and immunomodulator molecules. This research focused on characterizing the immune response of *C. virginica* to (a) bacterial pathogen *V. coralliilyticus* RE22 in order to understand mechanisms of pathogenesis in larval vibriosis and (b) bacterial probiotics *B. pumilus* RI06–95 and *P. inhibens* S4 in order to understand the role of immunomodulation in protection against RE22. Differential expression and functional enrichment analyses of oyster larvae 6 h after challenge with the bacterial pathogen *V. coralliilyticus* RE22 showed that larval oysters were able to recognize the pathogen, as indicated by upregulation of pattern recognition receptor genes *tlr4* and *tlr13*, but unable to mount an efficient immune response, evidenced by the downregulation of transcripts for several key genes in immune signaling pathways and the overall lack of expression in immune effector genes in response to RE22. This is in contrast with the responses of larval eastern oysters to probiotic treatment observed in our study (summarized in Figure 6), as well as results from previous studies in oysters and other bivalves showing a diversity of immune effectors, such as antimicrobial peptides (e.g., defensins), lysozymes, pore forming molecules like perforin, and serine protease inhibitors, produced in response to bacterial challenge (reviewed in [50]).

### 4.1. Pathogenesis of RE22 Vibriosis in Eastern Oyster Larvae: Role of Immunosuppression and Metabolic Stress

This research confirms the ability of RE22 to cause morbidity and mortality in eastern oyster larvae [14,16,51]. The dose of the pathogen RE22 used in these challenge experiments was similar to vibrio concentrations measured in temperate coastal environments during certain seasons and environmental conditions [9], and concentrations used in other challenge experiments performed for evaluation of transcriptomic responses in bivalves [20,52,53].

Our research suggests that targeted suppression of some immune pathways may be involved in the pathogenesis of vibriosis in larval oysters. In general, successful immune responses to gram negative bacteria, including vibrios, in oysters and other bivalves involve pathogen recognition by TLRs such as TLR13 and TLR4 (responding to 23S ribosomal RNA and LPS, respectively) (reviewed in [50]). Upon recognition of lipopolysaccharide (LPS) by TLR4, the receptor initiates the activation of the NF-kB and AP-1 (MAPK-dependent) pathways through recruitment of the adaptor molecule MyD88, currently the only known adaptor protein in bivalves, into the TIR domains of TLRs. Activation of TLR and interleukin-17 (IL-17) pathways ultimately leads to expression of immune effectors such as antimicrobial peptides and lysozyme, radical oxygen species (ROS) activation through respiratory burst, and expression of antioxidant genes. The NF-kB and MAPK signaling pathways also regulate apoptosis and inflammation (reviewed in [50,54]).

In eastern oyster larvae exposed to RE22, despite upregulation of *tlr4* and *tlr13*, genes for key immune signaling molecules that are usually upregulated in response to LPS in bivalves, such as *myd88*, *tak1*, and *nkap* [50,55] were instead downregulated, probably leading to the lack of expression in immune effectors regulated by the NF-kB signaling pathways seen in this study. Although downregulation of *ikb*⍺, an inhibitor of NF-kB, could potentially lead to transcription of immune effectors, changes in expression of these molecules are difficult to interpret, due to the complex feedback loop regulating NF-kB [55,56,57]. Another unexpected finding in this regard was the unique downregulation in vibrio but not probiotic-treated larvae of *tollip*, a negative immune regulator involved in the inhibition of pro-inflammatory responses to LPS [58]. Downregulation of *tollip* has also been reported 6 h after challenge of Yesso scallops, *Patinopecten yessoensis,* with *V. anguillarum* [59]. The function of Tollip in invertebrates is not well understood, but in vertebrates, it has been associated with a variety of signaling pathways, and its response to LPS is highly dependent on dose and context [60]. If TOLLIP has a similar function in oysters than in mammals, the downregulation of *tollip,* combined with the high levels of expression of *caspase1*, would be consistent with an inflammatory-like response in RE22 treated larvae, a response that has also been seen by histological examination [51]. In this sense, challenge of larvae with RE22 also led to an upregulation of gene expression for several molecules involved in the MAPK signaling pathway, including *mkk7* and *jak2*, suggesting that this pathway may not be directly targeted by RE22. Moreover, in addition to upregulation of the gene for TLR13 (an intracellular PRR involved in stimulation of expression of immune effectors through MyD88–NF-KB pathways; [61]), eastern oyster larvae exposed to RE22 showed upregulation of transcripts for STING, a key regulator for sensing intracellular single- or double-stranded nucleic acids that acts via the cGAS-STING pathway complex with TAK1 to trigger expression of interferon-related genes [62,63]. These results suggest that RE22 could be a facultative intracellular pathogen of larval oysters, as shown for other vibrio species affecting bivalves, such as *V. tasmaniensis* LGP32 [20,64]. Further functional research would be needed to identify the specific targets of RE22 immunosuppression in larval oysters, and its putative intracellular nature.

Evidence of host immunosuppression in response to pathogenic vibrios is in accordance with previous research in invertebrate hosts. Disturbance of host immune responses has been reported in adult Pacific oysters at early time points post challenge with virulent *Vibrio* sp., *V. splendidus* LGP32-GFP and *V. estuarianus* 02/041 [52], as well as *V. crassostreae* J2–9 [20]. Transcriptomic studies investigating the responses of coral *Pocillopora damicornis* to *V. coralliilyticus* YB1, which causes disease and bleaching in corals, also reported immunosuppression of the host [65,66]. Hemocytes of Mediterranean mussels, *Mytilus galloprovincialis,* are also unable to mount an efficient immune response towards *V. coralliilyticus* challenge, as shown by a lack of production of antimicrobial proteins and signs of lysosomal stress [18]. Moreover, while injection of sterile seawater into mussel tissues leads to expression of antimicrobial peptides (indicating that injection serves as a danger signal), mussels injected with *V. splendidus* do not show this upregulation in antimicrobial production [67]. These results, however, contrast with findings in scallop *Argopecten purpuratus* gills or adult giant clam *Tridacna crocea* hemocytes exposed to vibrio challenge, which showed upregulation of these immune pathways [68,69], suggesting that the immunosuppressive effects of vibrios on bivalve hosts depend on host and pathogen species and host developmental stage (adult *versus* planktonic larvae).

The inability of larval eastern oysters to mount a rapid and efficient immune response probably contributes to the rapid pathogen proliferation observed in larval tissues, accompanied by increased metabolic demand and signs of stress leading to heavy mortality seen within hours after challenge. Upregulation of *cox3, hspa12a,* and *hspa12b* is a key indicator of high metabolic demand and oxidative stress [70], and the lack of expression of genes coding for antioxidant enzymes suggests that larvae are unable to effectively handle the oxidative stress and high metabolic demand due to the inability to rapidly clear RE22 infection. This is consistent with the well-documented effect of bacterial infection on the transcriptomes of susceptible bivalve hosts [53,67,71,72,73].

### 4.2. Protection against RE22 Challenge in Probiotic-Treated Eastern Oyster Larvae: Role of Immunomodulation

This research also confirms the ability of probionts *B. pumilus* RI06–95 and *P. inhibens* S4 to protect eastern oyster larvae against challenge with RE22 when provided to the larvae at least 24 h before challenge [33,34]. In contrast to the oyster larvae response of RE22 challenge, exposure of larvae to probionts S4 and RI induced the expression of a variety of immune genes, suggesting a strong immune response comprising pathogen recognition through a variety of PRRs, activation of immune signaling pathways, and production of an arsenal of immune effectors. This probiotic mechanism of larval immunostimulation is consistent with our previous observations that probiotics are cleared from the larvae within 12–24 h after treatment [33]. These immune effectors activated in larvae upon probiotic exposure may also serve to provide protection against RE22 challenge, by leading to the production of immune effectors before RE22 has a chance to lead to immunosuppression in the host (Figure 5B–D).

Consistent with expectations based on the study of immunity against bacterial pathogens in bivalves and other species, as described in the section above, differential expression analysis in response to both probiotics suggests activation of various immune signaling pathways like TLR, NF-kB, and MAPK, leading to upregulation of transcripts for effectors involved in immune killing, cell proliferation, apoptosis and cell death, and the inflammatory response. Several probiotics are known to modulate (either activate or suppress) signaling pathways that benefit the host and protect them from pathogens [29,74,75,76,77]. Usually, probiotics show a very strain specific response in other host species [76,78]. In this case, however, despite the difference in Gram character between S4 and RI, many immune transcripts, especially effectors, were expressed in response to both probiotics. Our findings showing that *tlr3* (RI24h, S424h), *tlr4* (RI6h), *tlr6* (S424h), *tlr8* (RI24h), and *tlr13* (RI24h, S46h) were upregulated in response to probiotics are consistent with the important role of the TLR pathway in bivalve immune responses, and indicate the potential of probiotics to provide protection against a broad spectrum of pathogens. Such PRR activation by these probiotics due to shared cell envelope components such as lipopolysaccharides, peptidoglycans, and bacterial DNA with pathogens is well known [79].

Transcriptome analysis also suggests that activation of these pathways leads to increased transcription of a variety of immune effectors by probiotics that may have activity against vibrios and other pathogens. Perforin-2/Mpeg1, highly upregulated in larvae treated with either probiotic in the laboratory exposures, is an important ancient innate immune system effector that functions by forming pores in intracellular and extracellular pathogenic bacteria [80]. LPS exposure significantly upregulated a perforin-2 in oysters [81] and other invertebrates [82,83]. In addition to other functions, such as osmoregulation and responses to environmental stress, mucus is an important line of defense that plays multiple roles in the host-microbe interactions, including buffering pathogen secreted proteases and preventing invasion of pathogens through a vast array of immune recognition and effector proteins beneficial to the host that become embedded in the mucus layer [84,85,86,87]. All probiotic treatments showed highly upregulated serine protease inhibitors *Cvspi2* and *spi*, which might serve to neutralize serine proteases from RE22. Serine protease inhibitors like cvSI-1 have been shown to inhibit proliferation of the parasite *Perkinsus marinus* [88,89] and are upregulated in resistant eastern oysters in response to challenge with the pathogen *A. crassostreae* [72]. The presence of these immune effectors in larval tissues as a result of probiont RI and S4 pretreatment immediately prior to RE22 challenge may contribute to expedited clearing of the pathogen, alone or in combination with the ability of S4 to directly inhibit pathogen colonization and growth through antibiotic production, biofilm formation, and interference with quorum sensing in the pathogen [38,39].

Unique aspects of the HT_RI transcriptomes, which reflects continuous exposure in the hatchery to the probiotic versus a single exposure in the laboratory, was the upregulation of transcripts coding for histone H2B-like, GIMAP7, and several septins, and the enrichment of GO terms related to transport and localization of molecules/organelles/cells. Histones show antimicrobial action against Gram negative bacteria and in *C. gigas* has been demonstrated to surround and engulf vibrios [90,91,92]. GIMAP7 is a member of GTPase of the immune-associated proteins family that acts an apoptosis regulator [93]. Transcripts coding for various apoptosis inhibitors, as well as several caspases, were also differentially expressed in response to S4 and RI laboratory treatments, but these complex patterns are difficult to interpret due to the complexity of cell death pathways and the large expansion of genes involved in apoptosis seen in oysters [94]. In other organisms, autophagy and septins together restrict cytosolic bacterial replication [95]. Cytoskeletal rearrangements can help in bacterial sensing, compartmentalization, and phagocytosis of pathogens [96,97] as well as autophagy and apoptosis for host protection [98]. The role of these processes in immune responses in bivalves requires further research.

## 5. Conclusions

This study indicates that immunomodulation may contribute to the mechanisms of action of probionts RI and S4 against RE22 challenge, serving to overcome the immunosuppressive effects of RE22 on larvae. Additionally, differential gene expression analysis indicative of a high metabolic demand and oxidative stress are consistent with the rapid mortality observed during RE22 infection in oyster larvae. Further functional studies are required to tease out details of the mechanisms used by RE22 to manipulate the immune system of oyster larvae through targeting of immune signaling pathways. This study represents the first deep analysis of larval transcriptomes of eastern oysters in response to both bacterial pathogen and probionts, providing many candidate genes and specific pathways that should be targeted in the future for further characterization of mechanisms of *Vibrio* pathogenesis and probiotic immunomodulation. Based on this research, hatchery management solutions targeted to enhance the production of host immune effectors, such as immunostimulation by use of probiotics, should be evaluated as a cost-effective, natural, and environmentally-friendly solution for disease management.

## Figures and Tables

**Table 1:** Effect of probiotic pretreatment of oyster larvae for 6 or 24 h in the laboratory or daily for 7 days in the hatchery on larval survival after experimental challenge with the pathogen *V. coralliilyticus* RE22.

Treatment	RPS (Average +/− SD)		
	6 h	24 h	7 days
S4 + RE22	37 ± 26	41 ± 2	-
RI + RE22	30 ± 39	45 ± 5	28 ± 6

Note: Results are expressed as the relative increase in percent survival (RPS) +/− standard deviation (SD; *n* = 3 experiments) after RE22 challenge of larvae pretreated with probiotics (treatment) as compared to challenged larvae (control). S4 + RE22: Larvae pretreated with *Phaeobacter inhibens* S4 and then challenged with *V. coralliilyticus* RE22. RI + RE22: Larvae pretreated with *Bacillus pumilus* RI0-695 and then challenged with *V. coralliilyticus* RE22. —Not Tested.

## Supplementary Materials

The following are available online at https://www.mdpi.com/2076-393X/8/4/588/s1, Table S1: Depth of sequencing and alignment rates of oyster larval transcriptomes in response to pathogen and probiotic treatment; Table S2: Number of differentially expressed genes per comparison; Table S3–S8: Differentially expressed genes with log fold change for all treatments; Table S9: Mapping of differentially expressed transcripts to Gene Ontology (GO) terms for functional enrichment.
