# Supplementary material for: Contrasting Immunomodulatory Effects of Probiotic and Pathogenic Bacteria on Eastern Oyster, Crassostrea Virginica, Larvae
Source: Vaccines (Basel). 2020 Oct 6;8(4):588. doi: 10.3390/vaccines8040588 (PMC7720132; doi:10.3390/vaccines8040588)
Supplement: Supplementary file 1 [file vaccines-08-00588-s001.zip › Table S1.pdf]

Table S1. Depth of sequencing and alignment rates of oyster larval transcriptomes in response to pathogen and probiotic treatment.

Sample	Number of Paired Reads	% Alignment to *Crassostrea virginica* Genome
	**Laboratory Transcriptomes**	
C_K_0	22,963,376	89
C_M_0	16,617,375	88
C_V_0	20,674,506	86
RE_K_6	19,379,823	86
RE_M_6	21,118,821	89
RE_V_6	39,681,499	85
RI_K_24	27,507,148	86
RI_K_6	25,325,997	87
RI_M_24	22,339,707	86
RI_M_6	20,649,356	86
RI_V_24	18,412,447	83
RI_V_6	18,720,304	86
S4_K_24	25,285,770	87
S4_K_6	17,536,097	88
S4_M_24	21,950,812	87
S4_M_6	14,570,962	86
S4_V_24	17,840,669	89
S4_V_6	21,556,827	88
	**Hatchery Transcriptomes**	
HT_C_5d	73,690,654	53
HT_C_12d	60,768,394	93
HT_C_16d	70,771,125	64
HT_RI_5d	61,710,678	94
HT_RI_12d	59,865,884	94
HT_RI_16d	50,226,597	61

Laboratory transcriptomes: Oyster larvae were treated with *Vibrio coralliilyticus* RE22 for 6 h, *Bacillus pumilus* RI0–695 and *Phaeobacter inhibens* S4 for 6 or 24 h. Larvae for control (C) transcriptomes were collected at time 0 h. Hatchery transcriptomes: Larvae were treated daily in the hatchery with RI06–95 (RI) or not-treated (C), and collected 5, 12 or 16 d after fertilization. Three independent laboratory experiments (K, M, V) with three treatments (Control: C; RE22 treatment, RE, RI treatment: RI, S4 treatment: S4) were performed in duplicate. Number of paired end reads per sample and % alignment rates to *Crassostrea virginica* reference genome using HISAT2 are shown.
