# Supplementary material for: Contrasting Immunomodulatory Effects of Probiotic and Pathogenic Bacteria on Eastern Oyster, Crassostrea Virginica, Larvae
Source: Vaccines (Basel). 2020 Oct 6;8(4):588. doi: 10.3390/vaccines8040588 (PMC7720132; doi:10.3390/vaccines8040588)
Supplement: Supplementary file 1 [file vaccines-08-00588-s001.zip › Table S2.pdf]

Table S2. Number of differentially expressed genes per comparison ($p \leq 0.05$, upregulation: log fold change ≥2, downregulation: log fold change ≤ −2).

Comparison	Number of DEGs
**Lab Transcriptomes**	
RE22–6 h vs. C	1461
RI–6 h vs. C	1526
S4–6 h vs. C	2158
RI–24 h vs. C	1962
S4–24 h vs. C	2892
**Hatchery Transcriptomes**	
RI vs. C	2985

(Control: C; *Vibrio coralliilyticus* RE22 treatment: RE, *Bacillus pumilus* RI06–95 treatment: RI, *Phaeobacter inhibens* S4 treatment: S4).
